# MicroRNA-744 promotes prostate cancer progression through aberrantly activating Wnt/β-catenin signaling

**DOI:** 10.18632/oncotarget.14711

**Published:** 2017-01-18

**Authors:** Han Guan, Chunhui Liu, Fang Fang, Yeqing Huang, Tao Tao, Zhixin Ling, Zonghao You, Xu Han, Shuqiu Chen, Bin Xu, Ming Chen

**Affiliations:** ^1^ Department of Urology, Affiliated Zhongda Hospital of Southeast University, Nanjing, China; ^2^ Surgical Research Center, Institute of Urology, Medical School, Nanjing, China; ^3^ Department of Immunology, Bengbu Medical College, Bengbu, China

**Keywords:** miR-744, prostate cancer, Wnt/β-catenin signaling, NKD1, tumorigenesis

## Abstract

Accumulated evidence indicate that miR-744 functions as either tumor suppressor or oncogene in the progression of a variety of tumors, with a tumor type-specific way. However, little is known about how miR-744 impacts on the tumorigenesis of human prostate cancer. In this study, employing the analyses of microarray, qRT-PCR and re-analysis of MSKCC data, we found that CRPC tissues expressed much more miR-744 than ADPC tissues did, and the expression level of miR-744 was inversely associated with survival of CRPC patients. *In vitro* studies revealed that miR-744 promotes PCa cells proliferation, enhances migration, invasion; *in vivo* results demonstrated that silencing of miR-744 mediated by shRNA dramatically reduces PCa xenograft tumor growth. Importantly, through human gene expression array, pathway enrichment analysis and Western blot, we identified that miR-744 dramatically activated Wnt/β-catenin pathway by targeting multiple negative regulators of Wnt/β-catenin signaling, including SFRP1, GSK3β, TLE3 and NKD1. At molecular level, we further defined that NKD1 is a major functional target of miR-744. Our findings indicate that miR-744 acts as one of oncogenic factor in the progression of CRPC by recruiting a mechanism of aberrant activation of Wnt/β-catenin signaling.

## INTRODUCTION

As the most common malignancy and the second leading cause of cancer-related death among men in developed countries, prostate cancer (PCa) has long been considered as a substantial healthcare challenge in USA [1]. Recent years, with the economy improvement, the morbidity and mortality of PCa has also been steadily increased in China as well [2]. Even though the majority of PCa patients initially respond well for androgen deprivation therapy, the biggest hindrance for treatment of PCa is that most PCa patients within two years will inevitably progress to the castration-resistant prostate cancer (CRPC), a more aggressive form of PCa and the most common cause for prostate cancer patient death. The lacking of effective therapeutics strategies is the major limitation for treatment of recurrent and metastatic PCa [3]. Thus, it is urgent need to understand the molecular mechanisms underlying PCa progression and develop the novel promising therapeutic approaches.

MicroRNAs (miRNAs) are small, highly conserved non-coding RNA molecules which play the crucial roles in regulating diverse biological processes, such as proliferation, differentiation, apoptosis, development and metabolism [4, 5]. Accumulating evidence indicate that miRNAs may function as either oncogenes or tumor suppressors in the malignant progression of various cancers including prostate cancer [6–8]. For example, miR-34a has been demonstrated to function as a tumor suppressor to restrain PCa cells proliferation and metastasis of PCa stem cells whereas miR-21, miR-125b and miR-221, has been shown to serve as oncogenic factors to promote growth of PCa cells [9–12]. Until now, it has been discovered that more than 50 miRNAs are aberrantly expressed in PCa [13, 14], suggesting that deregulation of miRNAs is associated with tumorigenesis of this malignancy.

MiR-744 was initially identified in 2007, and a few years later miR-744 has been shown to serve as a tumor suppressor in several cancers including breast cancer, cervical cancer, colon cancer, and hepatocellular carcinoma [15–18]; on the other hand, miR-744 was highly expressed in head and neck cancer, pancreatic cancer, and nasopharyngeal carcinoma, and mediated the tumor-promotion effects on these cancers [19–23]. The contradictory effects of miR-744 on the various tumors indicate that miR-744 might exhibit its biological functions in tumor type-specific ways [15, 17]. In a previous miRNA microarray analysis, we have detected a panel of miRNAs are upregulated in CRPC clinical samples, including miR-744 [24]. However, the biological functions of miR-744 in tumorigenesis of human prostate cancer remain largely unknown.

In this study, we have systemically investigated the biological functions of miR-744 and its potential targets in the progression of PCa by utilizing a variety of approaches. Our results suggested that miR-744 serves as an oncogenic factor to promote PCa cells growth.

## RESULTS

### MiR-744 was overexpressed in CRPC and positively associated with CRPC progression

In our previous microarray analysis [24], we totally detected 1,646 miRNAs expressed in prostate cancer tissue. Among of them, we found 370 miRNAs were differentially expressed between ADPC (androgen-dependent prostate cancer) and CRPC. In the present study, we investigated those miRNAs that were overexpressed in CRPC with fold change > 1.5, comparing with that in ADPC. From the microarray dataset, we discovered 167 miRNAs were upregulated in CRPC, including miR-744, miR-3945, miR-1292 etc.

**Figure 1:**
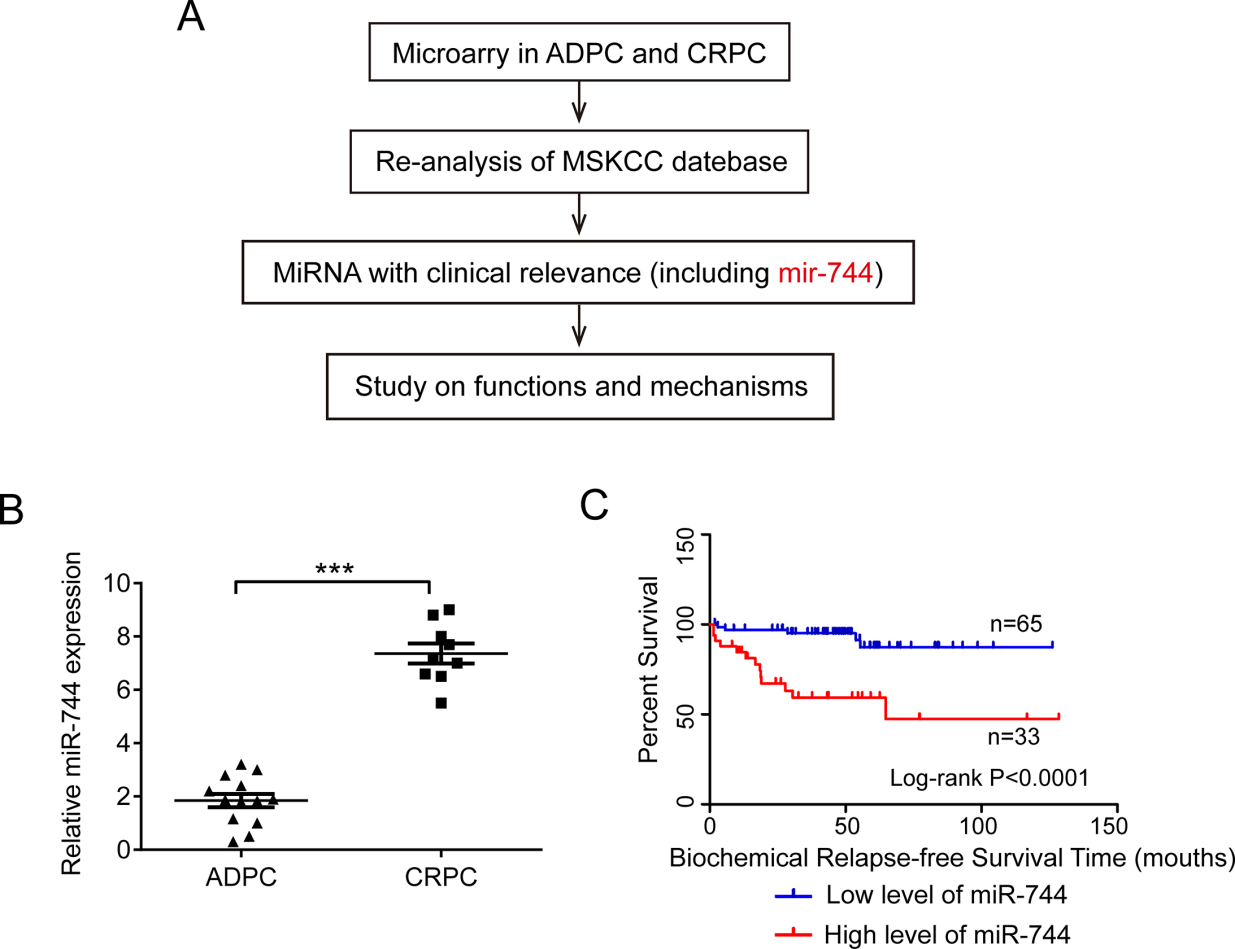
MiR-744 was overexpressed in CRPC and positively associated with CRPC progression (**A**) Experimental scheme. (**B**) Expression levels of miR-744 was verified by qRT-PCR in CRPC and ADPC tissues (*P* < 0.001). U6 RNA was measured as an internal control. (**C**) Kaplan–Meier analysis of biochemical relapse-free survival for 98 patients with PCa (Data acquired from MSKCC (GSE21032)). Patients with high miR-744 expression had a lower survival rate than those with low miR-744 expression (*P* < 0.0001).

To verify the microarray results, we performed quantitative real-time PCR (qRT-PCR) analysis on the expression levels of these miRNAs in 10 ADPC tissues and 10 CRPC tissues. Indeed, we found these miRNAs (such as miR-744, miR3945, miR-1292, miR-30c-1 and miR-4635) were significantly upregulated in CRPC samples compared to ADPC samples (*P* < 0.001, Figure 1B and Supplementary Table 1). Since miR-744 has been reported to play the critical roles in multiple other malignancies, but there is no report of miR-744 involved in human PCa. We therefore chose miR-744 to investigate its biological function in the progression of CRPC. We therefore chose miR-744 to investigate its biological function in the progression of CRPC (Figure 1A).

To validate whether the above conclusion is applicable to large number of clinical PCa samples, we conducted the re-analysis of the data acquired from MSKCC (GSE21032). As shown in Figure 1C, Kaplan-Meier analysis with the log-rank test revealed, after radical prostatectomy, that the biochemical relapse-free survival in the patients with low level of miR-744 was significant longer than that in the patients with high level of miR-744 (*P* < 0.0001). In order to clarify whether the miR-744 expression was associated with the outcome of PCa patients, we performed Cox regression analysis to confirm the variables of potential prognostic significance and the results suggested that the miR-744 expression (*P* = 0.006), Gleason score (GS) (*P* = 0.002), prostate-specific antigen (PSA) (*P* = 0.005) and lymph node invasion (LNI) (*P* = 0.002) were independent prognostic factors for biochemical relapse-free survival in patients with PCa. However, other factors such as seminal vesicle invasion (SVI), surgical margins (SMS), extracapsular extension (ECE) and pathological stage (pStage) showed no remarkable value in predicting prognosis (Supplementary Table 2). All results from MSKCC database implied that miR-744 represents a poor prognostic factor of CRPC patient.

Taken together, these results suggested that miR-744 function as an oncogenic factor in the progression of prostate cancer and its expression level is associated with the transformation of ADPC to CRPC.

### MiR-744 promotes PCa cells proliferation, migration, and invasion, and suppresses apoptosis *in vitro*

**Figure 2:**
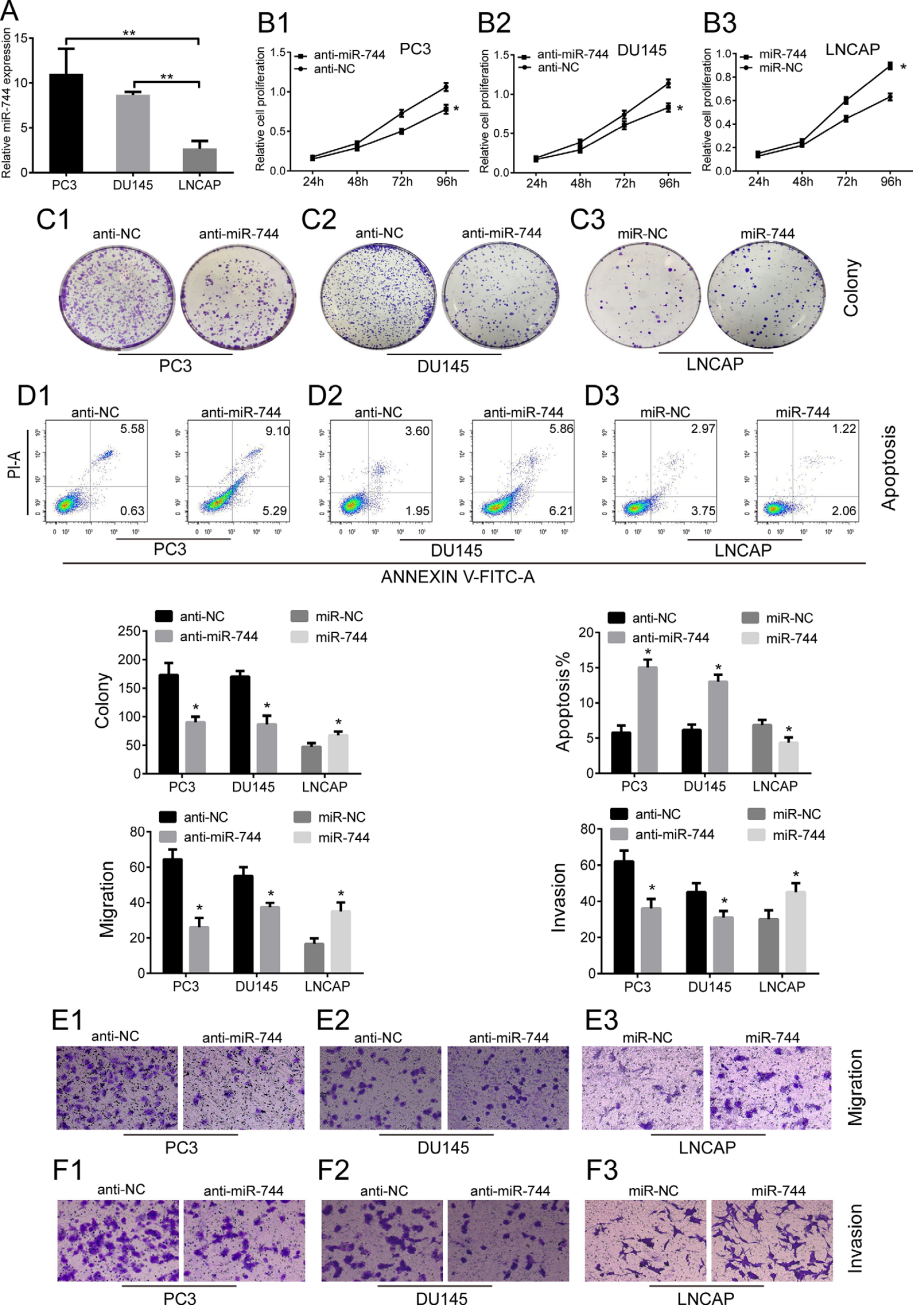
MiR-744 promotes PCa cells proliferation, migration, and invasion, but suppresses apoptosis *in vitro* (**A**) The expression of miR-744 was significantly up-regulated in CRPC cell lines (PC3 and DU145) than ADPC cell lines (LNCAP). (**P < 0.01). U6 RNA was measured as an internal control. (**B1**–**B3**) MTT assay showed that anti-miR-744 oligos (groups of anti-miR-744) suppressed growth rate in PC3 and DU145 cells while miR-744 minics (groups of miR-744) promoted growth rate in LNCAP cells. (**C1**– **C3**) Colony formation assay indicated that colony number of PC3 and DU145 cells transfected with anti-miR-744 oligos was lower than control, in contrast, the number of LNCAP transfected with miR-744 minics was higher than control. (**D1**–**D3**) Cell apoptosis assay. The consequence showed PC3 and DU145 cells with anti-miR-744 oligos demonstrate a higher apoptosis than control, on the contrary, the apoptosis in LNCAP cells transfected with miR-744 minics was lower than control. (**E1**–**E3**, **F1**–**F3)** The results of Transwell assay showed that migration and invasion ability of anti-miR-744 oligos group was lower than negative control in PC3 and DU145 cells, while cells with upregulated expression of mir-744 present a higher migration and invasion ability than control in LNCAP. Each bar represents the mean ± SD of three independent experiments. *P < 0.05.

To elucidate the mechanism of action of miR-744, we evaluate the impact of miR-744 on several biological properties of prostate cancer cells *in vitro*. Firstly, we performed qRT-PCR to determine the expression levels of miR-744 in different PCa cell lines. Consistent with the results in PCa tumor tissues, we found that miR-744 expression levels were dramatically increased in, we found that miR-744 expression levels were dramatically increased in CRPC cells (Du145 and PC3) and AIPC (androgen-independent prostate cancer) cells (LNCaP-AI) than in ADPC cells (LNCaP) (Figure 2A and Supplementary Figure 1A). Given that PC3, Du145 and LNCaP-AI cells expressed much more miR-744 than LNCaP cells did, we next assessed the effects of downregulation of miR-744 on cell growth of PCa cells. We transfected either anti-miR-744 oligos or anti-NC oligos into PC3, Du145 and LNCaP-AI cells, and examined cell proliferation by MTT assay, colony formation, cell death by Annexin V and PI staining. Compared to anti-NC oligos, we have observed that anti-miR-744 oligos not only greatly inhibited the cell growth of PC3, Du145 and LNCaP-AI cells (Figure 2B1, 2B2, 2C1, 2C2 and Supplementary Figure 1B, 1C), but also dramatically increased apoptosis of the three PCa cell types from 5.55% to 12.07% in Du145 cells, 6.21% to 14.39% in PC3 cells and 3.51% to 5.29% in LNCaP-AI cells (Figure 2D1, 2D2 and Supplementary Figure 1D). In contrast, when transfected synthetic miR-744 mimics in the miR-744-low expression LNCaP cells, we have observed that the enforced expression of miR-744 significantly enhanced LNCaP cells proliferation and suppressed the apoptosis of LNCaP cells (from 6.72% to 3.28%) (Figure 2B3, 2C3 and 2D3). These results demonstrated that miR-744 has the capacity to promote the PCa cell growth. We further asked whether miR-744 promotes the metastasis of PCa cells by performing experiments of transwell migration and invasion assay. As expected, anti-miR-744 oligos apparently inhibited the migration and invasion in PC3, DU145 and LNCaP-AI cells, compared with the anti-NC oligos (Figure 2E1, 2E2, 2F1, 2F2 and Supplementary Figure 1E, 1F). Correspondingly, overexpression of miR-744 mimics significantly enhanced the migration and invasion in LNCaP cells, compared with miR-NC transfection (Figure 2E3 and 2F3).

In summary, above results suggested, at cellular level, that miR-744 promotes PCa cell growth through enhancing cell proliferation, metastasis and reducing apoptosis.

### Reduction of MiR-744 suppresses the formation of prostate xenograft tumors *in vivo*

**Figure 3:**
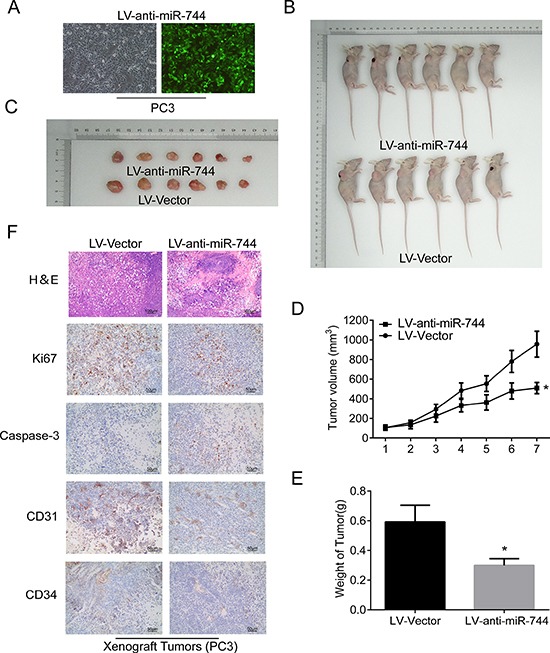
Reduction of MiR-744 suppresses the formation of prostate xenograft tumors *in vivo* (**A**) Fluorescence microscope is used for detecting transfection efficiency for LV-anti-miR-744 transfection and the results suggested transfection efficiencies are all more than 90%. (**B**) and (**C**) Subcutaneous tumors formed in nude mice by PC3 cells stably inhibition of miR744 or control at 28 days (*n* = 6/group). (**D**) Tumor formation growth curves after transfection of indicated cells. (**E**) Histograms describing the mean tumor weights of each group. Mean tumor volumes are plotted. (**F**) H&E and immunohistochemical staining of Ki67, activated caspase-3, CD31, and CD34 in the endpoint tumors revealed that reduced Ki67-positive cells, CD31-positive cells and CD34-positive cells, and significantly increased caspase-3-positive cells in miR-744 inhibitor-overexpressing PC3 tumors. Scale bars represent 50 μm and 100 μm. Each bar represents the mean ± SD of three independent experiments. *P < 0.05.

To determine whether miR-744 possesses tumor-promotion effects in PCa, we carried out xenograft tumor experiments in nude mice by monitoring tumor latency, incidence and endpoint weight. For this purpose, we constructed a lentiviral expression vector (LV-anti-miR-744) that encodes miR-744 inhibitor. By utilizing this expressing vector, we generated stably expressing-miR-744 inhibitor PCa cells by infected PC3 cells with the lentivius particles of LV-anti-miR-744 and corresponding negative control (Figure 3A), and subsequently implanted these infected PC3 cells into nude mice. As shown in Figure 3B–3E, silencing miR-744 by its inhibitor obviously suppressed tumor growth as manifested by reduced tumor size and tumor weight. In contrast, when endogenous miR-744 was stably overexpressed using LV-miR744 that encodes miR-744 mimic by infected LNCaP cells which expressed lower miR-744 than PC3 cells, the tumors were larger in size and had increased weight than those formed by corresponding negative control cells (Supplementary Figure 3A–3C).

Next, we performed H&E and immunohistochemical staining of Ki67, activated caspase-3, CD31, and CD34 in the endpoint tumors (Figure 3F). The results revealed that reduced Ki67-positive cells, CD31-positive cells and CD34-positive cells, and significantly increased caspase-3-positive cells in miR-744 inhibitor-overexpressing PC3 tumors. These data demonstrated that reduced miR-744 expression inhibits prostate tumor regeneration and growth by suppression of proliferation, angiogenesis, invasion and promotion of apoptosis. Furthermore, immunohistochemistry analysis also revealed that, compared to the control tumors, miR-744-overexpressing tumors had higher percentages of Ki-67–positive cells (Supplementary Figure 3D).

Altogether, the above experiments further confirmed that miR-744 serves as an oncogenic factor in tumorigenesis of prostate cancer.

### MiR-744 activates Wnt/β-catenin pathway by targeting multiple negative regulators and NKD1 is a crucial direct target of miR-744

**Figure 4:**
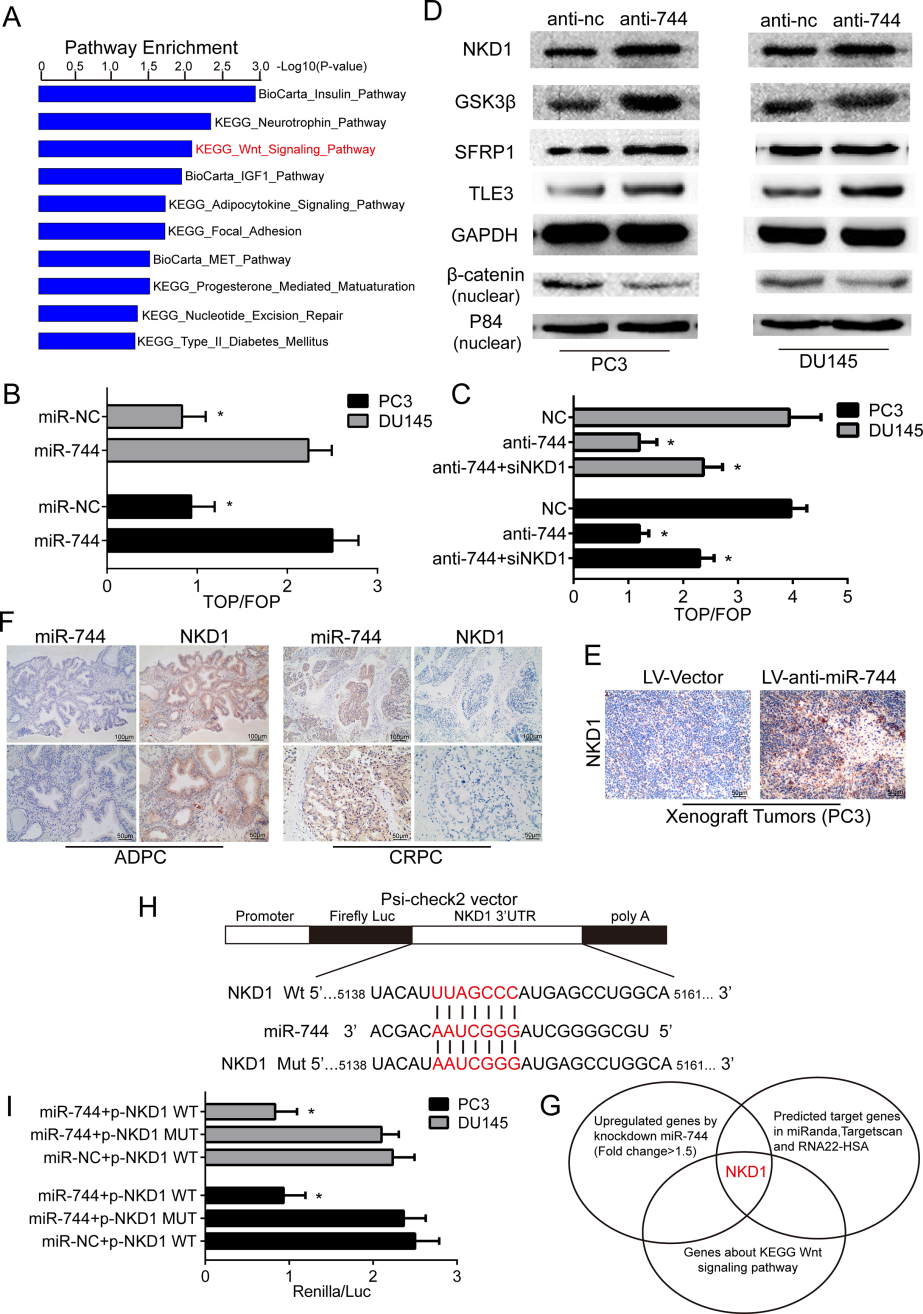
MiR-744 activates Wnt/β-catenin pathway by targeting multiple negative regulators and NKD1 is a crucial direct target of miR-744 (**A**) Top ten pathways sort by Kegg and Biocarta enrichment pathways result from array analysis. (**B**) Transfected with TOPflash or FOPflash and Renilla pRL-TK plasmids were subjected to dual-luciferase assays in PC3 and DU145 cells. Reporter activity detected was normalized by Renilla luciferase activity at 48h post-transfection. (**C**) Luciferase activity of TOP flash/FOP flash in the indicated cells. Each bar represents the mean ± SD of three independent experiments. *P < 0.05. (**D**) Protein expression of NKD1, GSK3β, SFRP1, TLE3 and nuclear β-catenin in both PC3 and DU145 cells was determined by Western blot assay after anti-mir-744 or anti-NC transfection. GAPDH was used as an internal control as well as P84 was served as an internal control in nucleus. (**E**) IHC staining with an anti-NKD1 antibody on PC3 xenograft tumors, we found that NKD1 positive cells in LV-anti-miR-744 treated PC3 xenograft tumors were much more than in the negative control tumors. (**F**) ISH and IHC staining on ADPC tissues and CRPC tissues with a miR-744 probe and an anti-NKD1 antibody, we observed that NKD1 expression was inversely correlated with miR-744 level. Scale bars represent 50 μm and 100 μm. (**G**) Overlap of miRNA target bioinformatic prediction methods, inhibition of mir-744 induced upregulated mRNAs and the negative regulators of Wnt signaling pathway. NKD1 was common to the three lists. (**H**) Principle scheme with RNA sequence alignment presented that the 3′-UTR of NKD1 mRNA contained a complementary site for the seed region of miR-744 (5138–5161bp) predicted by RNA22-HSA. I. The luciferase activity was detected that psiCHECK-2 luciferase reporter vector containing wild type and mutations of the binding sites in the 3′UTR of NKD1 mRNA with the miR-744 minics or miR-NC were co-transfected into PC3 and DU145 cells for 48 h. NKD1 mut was replaced the complementary region by a mutant as negative control. Rluc activity in the cells was measured and normalized to Fluc activity. Each bar represents the mean ± SD of three independent experiments. *P < 0.05.

To investigate the molecular mechanisms through which miR-744 exerts its prostate cancer-promoting effects, we conducted Affmetrix human gene expression array analysis on two transfected-PC3 cell lines that have been transfected with lentiviral constructs (LV-anti-miR-744 vs. LV-Vector to screen its potential targets. As a result, a total of 49,397 mRNAs were identified, however, only 214 of them were differentially regulated with fold changes ≤ 1.5 or ≥ 1.5 (LV-anti-miR-744 vs. LV-Vector including 86 down-regulated mRNAs and 128 up-regulated mRNAs. By employing KEGG and BioCarta pathways database, we found that Insulin signaling , Wnt signaling , IGF1 signaling and Focal Adhesion signaling are those of the top 10 enrichment pathways (Figure 4A). Interestingly, we detected that knockdown of miR-744 in PC3 cells significantly increased the expression of NKD1, a well-known negative regulator of Wnt signaling (Supplementary Table 3). These findings implied that Wnt signaling might be one major pathway involved in the progression of PCa mediated by miR-744.

As nuclear β-catenin is the critical effector of Wnt pathway, we therefore performed TOPflash/FOPflash luciferase reporter assay to determine whether the transcriptional activities of β-catenin will be enhanced in PCa cells when miR-744 is overexpressed. As shown in Figure 4B, the ratios of TOP/FOP in both PC3 and Du145 cells transfected with miR-744 mimics are significantly higher than in the two types PCa cells transfected with NC oligos. Futhermore, silencing NKD1 disrupted the repression efficacy of the miR-744-regulated Wnt/ β-catenin activity (Figure 4C), suggesting that miR-744 activates Wnt/β-catenin signaling through suppressing NKD1. This data suggested that enforced expression of miR-744 increases nuclear β-catenin activity and NKD1 is the key regulator for miR-744-induced Wnt/β-catenin activation. Moreover, Western blot analysis revealed that reduction of miR-744 not only enhanced NKD1 protein level, but also increased that the expression of other three negative regulators of Wnt signaling (GSK3β, SFRP1 and TLE3). Correspondingly, nuclear β-catenin levels in both PC3 and Du145 cells were reduced when miR-744 were depleted by anti-miR-744 oligos (Figure 4D). Because SFRP1 can directly bind Wnts protein and block Wnt pathways, and GSK3β can phosphorylate β-catenin and target β-catenin for proteasome-mediated proteolytic degradation, but NKD1 can interact with β-catenin in cytoplasm, therefore prevent its nuclear accumulation. Thus, our results indicate that miR-744 enhances Wnt signaling through suppression of different negative modulators of Wnt signaling in PCa cells. In addition, we examined the expression levels of negative regulators of Wnt/β-catenin signaling, particularly the expression level of NKD1 in LNCaP cells under both androgen-dependent and androgen-independent conditions. As shown in Supplementary Figure 1G, western blot analysis revealed that the expression of NKD1 GSK3β, SFRP1 and TLE3 were decreased in LNCaP-AI cells compared with LNCaP cells.

Given that above data suggested that NKD1 may be a target of miR-744 in PCa cells, we decided to investigate the relationship between NKD1 and miR-744 in PCa tumor samples. By conducted IHC staining with an anti-NKD1 antibody on PC3 and LNCaP xenograft tumors, we found that NKD1 positive cells in anti-miR-744 treated PC3 xenograft tumors were much more than in the negative control tumors (Figure 4E). Moreover, NKD1 positive cells in LNCaP xenograft tumors overexpressed miR-744 were lower than in the negative control tumors (Supplementary Figure 3D). Strikingly, when we performed ISH and IHC staining on 10 ADPC tissues and 10 CRPC tissues with a miR-744 probe and an anti-NKD1 antibody, we observed that NKD1 expression was inversely correlated with miR-744 level (Figure 4F). Above results, together with several pieces of evidence that NKD1 is a well-known negative regulator of Wnt signaling in a variety of tumor types, we therefore hypotheses that NKD1 is a main direct regulators of miR-744.

Thus, we next used three bioinformatics tools (TargetScan, miRANDA, RNA22-HSA) to search whether NKD1 is one of putative targets of miR-744. Indeed, the prediction analysis revealed that NKD1 is a direct target of miR-744 since the 3′-UTR of NKD1 gene contains a binding site that perfectly complements with the seed sequence of miR-744 (Figure 4G). To verify whether NKD1 gene is a functional target of miR-744, we carried out the luciferase report assay by co-transfecting miR-744 mimics with psi-CHECK-NKD1-WT (harbors the wild-type miR-744 binding site in NKD1 3′-UTR downstream of the firefly luciferase gene) or psi-CHECK-NKD1-MUT (contains a mutated miR-744 binding site in NKD1 3′-UTR) into Du145 and PC3 cells (Figure 4H). As our expectation, co-transfection of the two cell lines with the luciferase construct (wild type) and miR-744 mimic led to reduce luciferase activity significantly. Mutation of the miR-744 binding site in the NKD1- 3′-UTR abrogated this miR-744 effects (Figure 4I), testifying NKD1 as a direct target of miR-744.

Altogether, above results clearly indicate that miR-744 activates Wnt/β-catenin pathway by targeting multiple negative regulators and NKD1 is a main functional target of miR-744 in prostate cancer development.

### NKD1 is a critical downstream mediator of miR-744 effects prostate cancer progression

**Figure 5:**
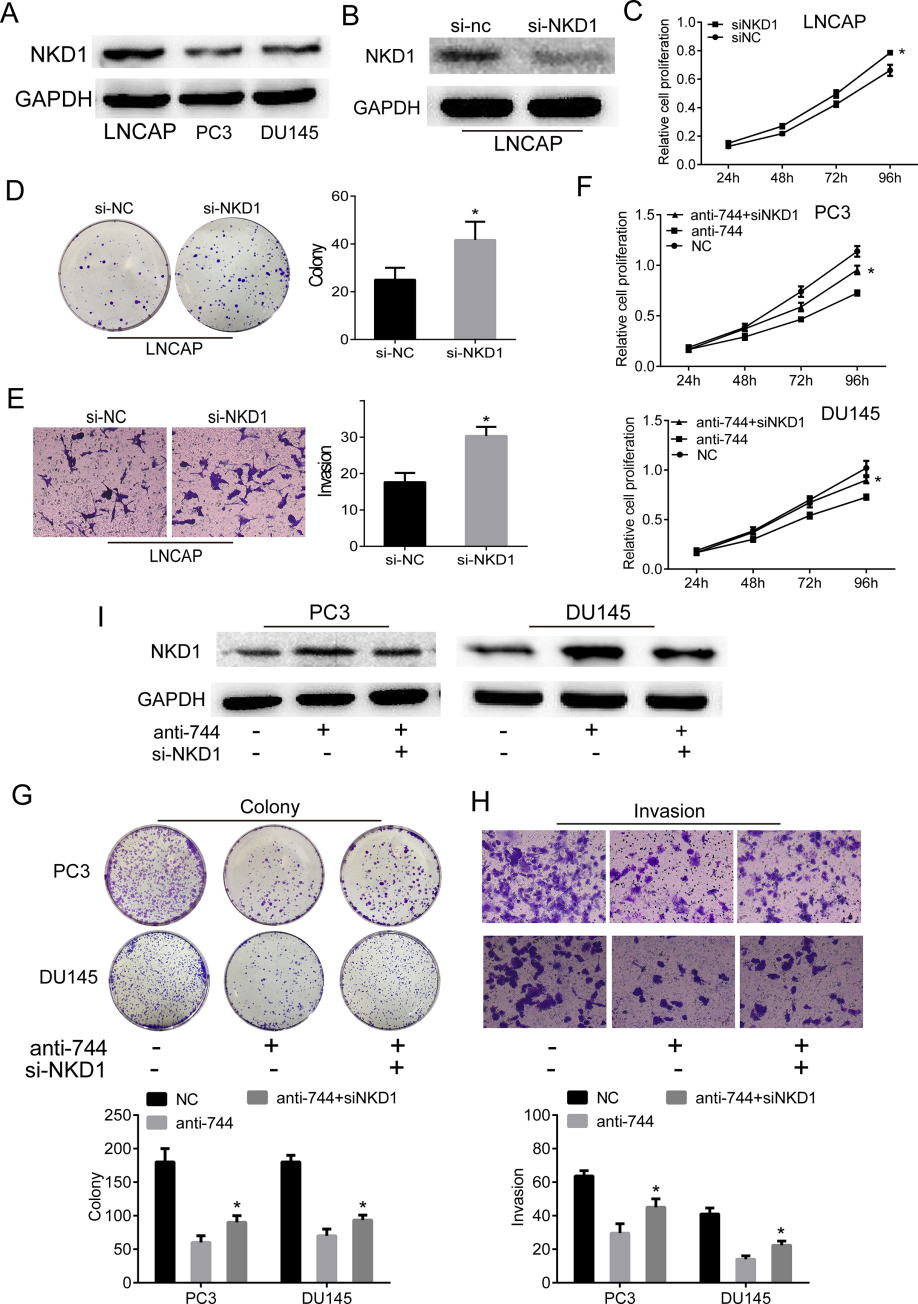
NKD1 is a critical downstream mediator of miR-744 effects in prostate cancer progression (**A**) Compared with ADPC cell lines (LNCAP), lower expressions of NKD1 protein were found in CRPC cell lines (PC3 and DU145) by western blot. (**B**) Western blot confirmed that protein of NKD1 in LNCAP cells after transfected with siRNA-NKD1 was lower than transfected with siRNA-NC. GAPDH was used as an internal control. (**C**–**E**) Reduction of NKD1 by siRNA knockdown in LNCAP cells markedly inhibited cell proliferation and colony formation as well as invasive abilities, compared to the siNC-treated cells. (**F**–**H**) Knockdown of NKD1 moderately attenuated the inhibitory effects on cell proliferation, colony formation, migration and invasion of PCa cells induced by inhibition of miR-744 level. Each bar represents the mean ± SD of three independent experiments. *P < 0.05. I. Western blot assay demonstrated that siRNA-mediated downregution of NKD1 apparently antagonized the enhancement of NKD1 protein induced by anti-miR-744 in both Du145 and PC3 cells. GAPDH was used as an internal control.

To provide the further evidence that NKD1 is a direct downstream mediator of miR-744, we first detected the expression of NKD1 between these PCa cells and found that the expression of NKD1 in PC3 and DU145 cells were less than LNCaP cells, then we conducted the siRNA-mediated NKD1 knockdown experiments on LNCaP cells (Figure 5A and 5B). As shown in Figure 5C–5E, reduction of NKD1 by siRNA knockdown in LNCaP cells markedly inhibited cell proliferation and colony formation as well as invasive abilities, compared to the siNC-treated cells. In contrast, overexpression of NKD1 in PC3 and DU145 cells significantly promoted cell proliferation, colony formation and invasive abilities, compared to the empty-vector infected cells (Supplementary Figure 2A–2D). These results, together with the facts that lentirival downregulated miR-744 suppressed and upregulated miR-744 promoted the xenograft tumor growth in nude mice (Figure 3 and Supplementary Figure 3) and luciferase report assay (Figure 4), suggested that miR-744 exerts the capacity of tumor promotion by targeting NKD1. To examine whether NKD1 is a functional important target of miR-744, we performed “antagonistic effects” experiments by co-transfecting Du145 and PC3 cells with sh-anti-miR-744 and siNKD1. The results in Figure 5F–5H, revealed that knockdown NKD1 by siRNA moderately attenuated the inhibitory effects on cell proliferation, colony formation, migration and invasion of PCa cells induced by reduction of miR-744 level. Western blot analysis also demonstrated that siRNA-mediated downregution of NKD1 apparently antagonized the enhancement of NKD1 protein induced by anti-miR-744 in both Du145 and PC3 cells (Figure 5I).

Thus, above experiments demonstrated that NKD1 is a critical downstream mediator of miR-744 effects in prostate cancer progression.

## DISCUSSION

In this study, we provided the convincing evidences for the conclusion that miR-744 behaved as an oncogenic factor in the progress of prostate cancer, especially in the transition from ADPC to CRPC. The expression level of miR-744 in CRPC samples was much higher than in ADPC samples, and its expression level is inversely associated the survival of CRPC patients. From the analyses of *in vitro* and *in vivo*, we observed that miR-744 exhibited its abilities to promote cell proliferation, enhance the abilities of migration, and inhibit apoptosis. Importantly, we have identified that miR-744 downregulated the expressions of multiple negative modulators of Wnt signaling, particularly the expression of NKD1, thereby aberrantly activated Wnt/β-catenin pathway, as the consequence, promoted the progression of CRPC.

Recently, the deregulation of miR-744 has been frequently observed in many types of cancers. It has been reported that miR-744 not only highly expressed in head and neck cancer, pancreatic cancer and nasopharyngeal carcinoma, and promoted tumor growth in these cancers, but miR-744 also exhibited an inhibitory effect on the progression of breast cancer, cervical cancer, colon cancer and hepatocellular carcinoma. All these findings imply that miR-744 could function either as oncogene or as tumor suppressor in a cellular context-dependent manner. Even though Huang et al. reported that short-term expression of miR-744 enhanced the proliferation of mouse prostate adenocarcinoma cells whereas long-term expression of miR-744 suppressed tumor growth *in vivo* [25]. In addition, Hatano K et al. demonstrated that miR-744-3p inhibit DNA repair and sensitize prostate cancer cells to ionizing radiation [26]. However, the exact biological function of miR-744 on development of human prostate cancer has not been reported. In our current study, we discovered that miR-744 levels are much higher in human CRPC specimens than in human ADPC samples, and its high expression was positively correlated with the advanced stage and poor prognosis of CRPC patients. Further, we found that miR-744 dramatically promoted cell proliferation, migration and invasion of CRPC *in vitro*, as well as enhanced xenograft tumor growth *in vivo*, indicating that miR-744 behaves as an oncogenic factor in the progression of ADPC to CRPC.

MiR-744 exhibits its functions on the progression of a variety of tumors by employing multiple molecular mechanisms. For example, by down-regulating Bcl-2, miR-744 enhanced apoptosis and inhibited cervical cancer cell proliferation; in hepatocellular carcinoma miR-744 exerted tumor suppressor function by targeting c-Myc [15]; miR-744 was reported to promote the progression of nasopharyngeal carcinoma via directly interacting with the promoter region of ARHGAP5 [20]. Among the involved mechanisms, the activation of Wnt/β-catenin signaling by miR-744 might represent more clinical significance.

Wnt signaling is a critical pathway for regulating development and adult tissue homeostasis, and β-catenin is a key effector of Wnt signaling. In absence of Wnt stimulation, β-catenin mainly exists in cytoplasm and will be degraded via ubiquitin-proteasome mechanism. After Wnt pathway is activated, β-catenin will be released from the “destruction complex” which comprised of adenomatous polyposis coli (APC), casein kinase1α (CK1α), glycogen synthase kinase 3β (GSK3β) and Axin. This release of β-catenin leads to its nucleus translocation and interaction with TCF/LEF transcription factors, thus activating the transcription of multiple target genes [27, 28]. However, aberrant activation of Wnt signaling will induce the accumulation of nuclear β-catenin, resulting in constitutively transcriptionally activation of proto-oncogenes related with cell proliferation and apoptosis [29].

Aberrant activation of Wnt/β-catenin signaling has been frequently identified in many tumor types such as colorectal, liver, lung and ovarian cancer etc [30]. For prostate cancer, up-regulation of Wnt/β-catenin has been shown to promote PCa progression *in vivo* and is correlated with high Gleason score, hormone-refractory and metastatic PCa status [31, 32]. Therefore, the activity of the Wnt pathway needs to be regulated delicately to maintain its proper function during development and in adult tissue homeostasis. Until now, increasing evidences have demonstrated that multiple negative modulators could antagonize Wnt/β-catenin signaling through recruiting different mechanisms. For example, secreted Frizzled-related proteins (SFRPs) and Wnt inhibitory factor-1 (WIF-1) are able to directly bind Wnt proteins and block Wnt/ β-catenin pathway, and Dickkopf1 (DKK1) negatively regulates Wnt signaling via interacting with Wnt co-receptors LRP5/LRP6 whereas GSK3β displays its suppression on Wnt/β-catenin pathway through phosphorylating β-catenin, leads to the proteasome proteolytic degradation of β-catenin [28, 33, 34]. Moreover, nuclear transcriptionalsuppressors including transducin-like enhancer of split 3 (TLE3) inhibit the transcriptional activity of LEF/TCF [35]. Intriguingly, a number of recent studies implied that NKD1 could interact with β-catenin in cytoplasm and interrupt the nuclear accumulation of β-catenin, thereby repressing the aberrant activation of Wnt/β-catenin pathway [36, 37]. Thus, targeting and regulating these negative modulators will represent a novel strategy to control the aberrant activation of Wnt/ β-catenin signaling. In the present study, we identified that Wnt/ β-catenin pathway as the one of top ten pathways involved in the progress of CRPC by utilizing pathway enrichment analysis. And in PCa cells, we not only observed that miR-744 inhibited the expression of SFRP1, a well known secreted negative regulators, and but also down-regulated significantly the production of GSK3β and TLE3. Most importantly, we identified that miR-744 dramatically reduced the expression of NDK1 protein in CRPC cells. At molecular level, by employing the analysis of microarray, Western blot, bioinformatics tool and luciferase reporter assay, we further confirmed that miR-744 could directly targeted the 3′-UTR region of NKD1 transcripts, which abolished the blocking effect of NKD1 on the nuclear accumulation of β-catenin, therefore greatly activates Wnt/β-catenin pathway. Thus, our results uncover a novel mechanism that the tumor-promotion effects of miR-744 on the progression of CRPC are accomplished by disrupting three different layers of negative regulations of Wnt/β-catenin signaling. Consistent with our result, the findings from Zhou's study also demonstrated that high level of miR-744 displayed the capacity to aberrantly activate Wnt/β-catenin signaling by directly suppressing the production of three negative regulators of Wnt/β-catenin pathway (SFRP1, GSK3β and TLE3), resulted in promoting the carcinogenesis of pancreatic cancer [23]. These conclusions indicate that miR-744 may represent a potent novel targets for development of new therapeutic strategy for treatment of CRPC and pancreatic cancer.

In the progression of ADPC to CRPC, reactivation of AR pathway is a key molecular event [38]. It has been reported that increase expression and nuclear co-localization of AR and β-catenin occur in CRPC [39]. Moreover, AR expression and Wnt/β-catenin activation correlate with aggressiveness and metastatic status in PCa patients [40, 41]. Thus, it would be of great interest to further explore the relation between upregulation of miR-744 and reactivating of AR pathway in the progress of in CRPC.

## MATERIALS AND METHODS

### Memorial sloan kettering cancer center (MSKCC) prostate cancer database re-analyses

We derived the original microRNA expression data and related clinical data from MSKCC prostate cancer database (GSE21032) and performed the re-analyses including the expression of miR-744 in different prostate cancer stages, mainly based on Gleason scores, and the correlation between miR-744 expression levels and biochemical relapse-free time of patients after radical prostatectomy.

### Patient samples

ADPC tissues were obtained from ten patients that underwent radical prostatectomy and never received any previous treatment. Among the patients, eight patients were diagnosed with ADPC stage II and two patients with stage III; seven patients had Gleason score < 7; two patients with Gleason score = 7, and one patient with Gleason score > 7. CRPC specimens from ten patients who were diagnosed with CRPC, since their serum prostate-specific antigen (PSA) levels continued to increase maxima during androgen-deprivation therapy. CRPC patients were all in stage IV and had a Gleason score > 8. The clinical samples were obtained during transurethral prostatic resection (TURP) because of urinary retention. For each specimen, a portion of tumor tissue was confirmed as staining for PSA and only the samples with > 60% tumor involvements were included in the study. Three paired tissues of ADPC and CRPC in each group were snap frozen in liquid nitrogen for the microarray analysis; other tissues were used for real-time PCR confirmation. All of the samples were obtained with the patients’ informed consent and with approval from the Ethics Committee of the Affiliated Zhongda Hospital of Southeast University.

### Cell culture

LNCaP, Du145, and PC3 were obtained from ATCC. LNCaP and Du145 cells were maintained in the medium of RPMI-1640 (Gibco, Thermo Fisher Scientific, USA) supplemented with 10% fetal bovine serum and antibiotics; PC3 cells were cultured in DMEM/12 medium (Gibco, Thermo Fisher Scientific, USA) supplemented with 10% fetal bovine serum and antibiotics. Cells from passage 8 to 15 were used in the experiments. An androgen-independent subline form LNCaP cells (LNCaP-AI) was established by continuously culturing the LNCaP cells in their regular medium. The cell culture medium was changed to a phenol red-free RPMI 1640 with 10% fetal bovine serum which was depleted of steroids by charcoal/dextran-treatment (CDS) (Biological Industry, Israel) instead of regular fetal bovine serum. Cells from passage 12 to 18 were used in the experiments [42]. The cells were cultured at 37°C in 5% CO_2_.

### RNA extraction and quantitative real-time PCR (qRT-PCR)

Total RNA was extracted from cells with TRIzol (Invitrogen™, Thermo Fisher Scientific, USA). qRT-PCR was carried out by using Fermentas reverse transcription reagents and SYBR Green PCR Master Mix of Hairpin-it™ miRNAs RT-PCR Quantitation Kit (GeneChem, Shanghai, China) according to manufacturer's instruction. The expression of U6 was used as a control. The primers for miR-744 amplification were as follow: miR-744 Forward primer: 5′-ACACTCCAGCTGGGT GCGGGGCTAGGGCTAAC-3′; U6 Forward primer: 5′-GCTTCGGCAGCACATATACTAAAAT-3′; Uni-miR Reverse primers: 5′-CTCAACTGGTGTCGTGGA-3′. PCR were conducted on ABI 7300 system (Applied Biosystems, USA) and relative gene expression was calculated by the 2^−ΔΔCt^ method.

### Oligonucleotides, plasmids, and luciferase reporter assays

Based on miRBase database, miR-744 mimic, negative control of miRNA (miR-NC), anti-miR-744 oligos (anti-miR-744) and negative control anti-miRNA (antiNC) were designed and synthesized by GeneChem (Shanghai, China). Their sequences were as follow:

(1) miR-744 mimic sense: 5′-UGCGGGGCUAG GGCUAACAGCA-3′; (2) miR-744 mimic antisense: 5′-CUGUUAGCCCUAGCCCCGCAUU-3′; (3) miR-NC sense: 5′-UUCUCCGAACGUGUCACGUTT-3′; (4) miR- NC antisense: 5′-ACGUGACACGUUCGGA GAATT-3′; (5) anti-miR-744 oligos: 5′-UGCUGUUAGCCCUAGCCC CGCA-3′; (6) anti-miR-NC: 5′-CAGUACUUUUGUGUA GUACAA-3′;

NKD1 siRNA (siNKD1) and negative control siRNA (siNC) were all purchased from Santa Cruz Biotechnology (Santa Cruz, USA). The cells were transfected with the aforementioned siRNA for 48h using Lipofectamine 2000 (Life Technologies, USA) following the manufacturer's protocol. The efficiency of knockdown was determined by western blot analysis. The reporter vectors containing wild type (CCTTTGATC; TOP flash) or mutated (CCTTTGGCC; FOP flash) TCF/LEF DNA binding sites were purchased from Upstate Biotechnology (New York, USA). The fragments of NKD1 3′-UTR containing either putative miR-744 seed sequence (wild-type, 5′-TACATTTAGCCCATGAGCCTGGC-3′) or mutated seed sequence (mutant, 5′-TACATAATCGGG ATGAGCCTGGC-3′) were synthesized by GeneChem (Shanghai, China). We subcloned wild type and mutant NKD1 3′-UTR into psiCHECK-2™ vector (Promega, USA) to obtain reporters of psi-CHECK-NKD1-WT and psi-CHECK-NKD1-Mut. Prostate cancer cells were seeded in 24-well plates and co-transfected them with reporters and miR-744 mimics and miR-NC together with Renilla luciferase internal normalization plasmid (phRL-CMV). We determined the ratio of firefly to Renilla luciferase activity with a dual luciferase assay (Promega, USA) 48 h later.

### Stable transfection

Reverse complement sequence of miR744 mature was synthesized and subcloned into the AgeI/EcoRI site of GV280 vector (GeneChem, Shanghai, China) to generate the construct that inhibits miR744 expression, and this construct was named LVanti-miR744. The GFP vector was used for control. PC3 cells were transfected with LVanti-miR744, or control vector. Conversely, LNCaP cells stably overexpressing miR-744 vectors (GV209) or control vector constructed by GeneChem (Shanghai, China) were established by infection with lentivirus named LV-miR-744. Lentiviruses carrying overexpressing human NKD1 lentiviral vectors (GV358) were from GeneChem. The viruses were used to infect cells in the presence of Polybrene. After 48 hours, cells were cultured in medium containing puromycin for the selection of stable clones. The efficiency of knockdown and overexpression were determined by realtime quantitative reverse transcription-polymerase chain reaction (qRT-PCR) and western blot analysis. The GFP vector was used for control.

### Microarray processing and analysis

Total RNA from PC3 cells infected with lentivirus expressing either LV-anti-miR-744 or LV-Vector was extracted using Trizol reagents. Then RNA quantity and quality were assessed with NanoDrop 2000 and Agilent Bioanalyzer 2100. And Affymetrix human GeneChipprimeview was used for microarray processing to determine gene expression profiling depending on the manufacturer's instructions. In brief, reverse transcription, double-stranded DNA template conversion, *in vitro* transcription for mRNA synthesis and labelling were all performed using GeneChip 3′IVT Expression Kit (Affymetrix, USA). Microarray hybridization, washing, and staining were then performed using GeneChip Hybridization Wash and Stain Kit (Affymetrix, USA). Arrays were then scanned using GeneChip Scanner 3000 to produce raw data. Significant differentially expressed genes between PC3 cells treated with LV-anti-miR-744 and LV-Vector were selected based on the following criteria: *P* value < 0.05 and absolute fold changes ≤ 1.5 or ≥ 1.5. Pathway enrichment analysis was performed for all significant differential genes based on KEGG and BIOCARTA pathway databases.

### Western blot analysis

Cells were lysed in RIPA buffer (25 μM Tris-HCl (pH 7.6), 150 μM NaCl, 1% NP-40, 1% sodium deoxycholate, 0.1% SDS) supplemented with 1% proteinase inhibitors (Cat. No: P2850, Sigma) for total protein preparation. Nuclear protein extracts were obtained with Nuclear Extraction Kit (Active Motif) according to the manufacturer's instructions. Briefly, 40 μg protein samples were analyzed by 10% SDS-PAGE and the gels were transferred onto a polyvinylidene fluoride membrane. The membranes were blocked with 5% non-fat dried milk in TBST (10 mM Tris-HCl, 150 mM NaCl and 0.1% Tween-20) for 1 h at room temperature, and incubated overnight at 4°C with specific primary antibodies. Membranes were washed three times with TBST buffer, then incubated for 1 h with 1:2000 secondary antibodies, and developed with enhanced chemiluminescence (ECL, Millipore, USA). The primary antibodies included anti-NKD1 (1:1000, Abcam, USA), anti-SFRP1 (1:1000, Abcam, USA), anti-GSK3β antibody (1:1000, Abcam, USA), anti-TLE3 antibody (1:1000, Abcam, USA) and anti-β-catenin (1:1000, Abcam, USA) antibody. Anti-GDPAH (1:5000, Beyotime, China) and anti-p84 (1:1000, Abcam, USA) antibodies were used as the loading control.

### Apoptosis assay

Apoptosis was conducted with Annexin V-FITC/Propidium Iodide (PI) Apoptosis Detection Kit (Beyotime, China). After transfection, prostate cancer cells were stained with 10 ul VFITC and 10 ul PI and cultured in the dark at room temperature for 15 minutes. The treated cells were analyzed by flow cytometry. Cell Quest Pro Software (BD Biosciences, CA) was used to analyze the cell apoptosis.

### MTT and transwell assays

For MTT assays, 2,000 cells were seeded in 96-well plates and transfected with various vectors for 24 to 96 hours using Lipofectamine 2000 (Life Technologies, USA). Then, the cells were stained with 100 μL MTT dye (Beyotime, China) for 4 hours at 37°C, removed the supernatant and followed by adding 50 μL dimethyl sulphoxide (DMSO). The optical density was measured at 570 nm with a microplate reader (Bio-Tek, USA). For invasion assays, PCa cells were transfected with miR-744 or anti-miR-744 oligos for 48 hours, after which 50,000 cells in serum-free medium were seeded in the top chamber of 24-well transwell units which was precoated with Matrigel (BD Pharmingen, USA) with RPMI-1640 containing 10% FBS added to the bottom chambers. Cells were allowed to migrate for 24 hours at 37°C, and then cells in the top chambers were removed, and cells that invaded into the bottom chambers were fixed, stained with 1.0% crystal violet. The cells on the bottom chamber of the membrane were manually counted and photographed by using an inverted microscope (×200 magnification).

### Colony formation assay

In brief, 800 cells were seeded in 6-well plates and incubated for 12 days at 37°C in 5% CO_2_. Next, the cells were washed twice with phosphate buffer solution, fixed with methanol for 15 min, and stained with 0.2% crystal violet for 20 min at room temperature. Colonies containing more than 50 cells were counted.

### Tumorigenicity assay *in vivo*

BALB/C nu/nu female mice (6 weeks) were purchased from the Shanghai SLAC Laboratory Animals. All animal experiments were conducted according to the National Institute of Health Guide for the Care and Use of Laboratory Animals and were approved by the ethics committee of the Affiliated Zhongda Hospital of Southeast University. PC3 (4 × 10^6^) cells and LNCaP (8 × 106) cells that have been stably transfected with LV-anti-miR744 and LV-miR-744 were inoculated subcutaneously together with Matrigel into the oxter flank of nude mice. After 7 days of implantation of tumor cells, tumor size was measured every 4 days and tumor volumes were calculated with the following formula: (length × width^2^)/2. At the end point of experiments, the mice were sacrificed and tumors were dissected and weighed.

### *In situ* hybridization (ISH) and immunohistochemical staining (IHC)

ISH and IHC were performed as previously described. In brief, the double (5′–3′) digoxigenin (DIG)-labeled miR-744 probe and U6 probe were purchased from Boster (Wuhan, China) and ISH was conducted according to the manufacturer's instructions of the microRNA ISH Optimization Kit (Boster, Wuhan, China). IHC was carried out with appropriate primary antibodies according to their manufacturer's instructions. These antibodies included anti-NKD1 (1:100, Abcam, USA), anti-ki67, anti-caspase-3, anti-CD31, and anti-CD34 (All 1:100, Boster, China). ISH and IHC scores were performed using a semiquantitative grading system as our previous study [43]. Sections with no labeling or with fewer than 5% labeled cells were scored as 0. Sections with 5%–30% of cells labeled were scored as 1, with 31%–70% of cells labeled as 2, and with labeling of ≥ 71% as 3. The staining intensity was scored similarly, with 0 used for negative staining, 1 for weakly positive, 2 for moderately positive, and 3 for strongly positive. Each sample was examined separately and scored by two pathologists. Cases with discrepancies in the scores were discussed to reach a consensus.

### Statistical analysis

MiR-744 expression with clinical patient data was downloaded from the MSKCC database (http://www.mskcc.org). All statistical analyses were performed with the Statistical Package of the Social Sciences software version 16.0 (SPSS, Inc.). All experiments above were repeated three times and statistical analyses were utilized a two-tailed Student's *t*-test. Data are presented as means and standard deviation (SD). Statistical significance was set as *P* < 0.05. *P < 0.05, **P < 0.01, ***P < 0.001

## SUPPLEMENTARY MATERIALS FIGURES AND TABLES


