# Supplementary material for: MicroRNA-744 promotes prostate cancer progression through aberrantly activating Wnt/β-catenin signaling
Source: Oncotarget. 2017 Jan 18;8(9):14693–707. doi: 10.18632/oncotarget.14711 (PMC5362436; doi:10.18632/oncotarget.14711)
Supplement: Supplementary file 1 [file oncotarget-08-14693-s001.pdf]

MicroRNA-744 promotes prostate cancer progression through aberrantly activating Wnt/β-catenin signaling

Supplementary Materials

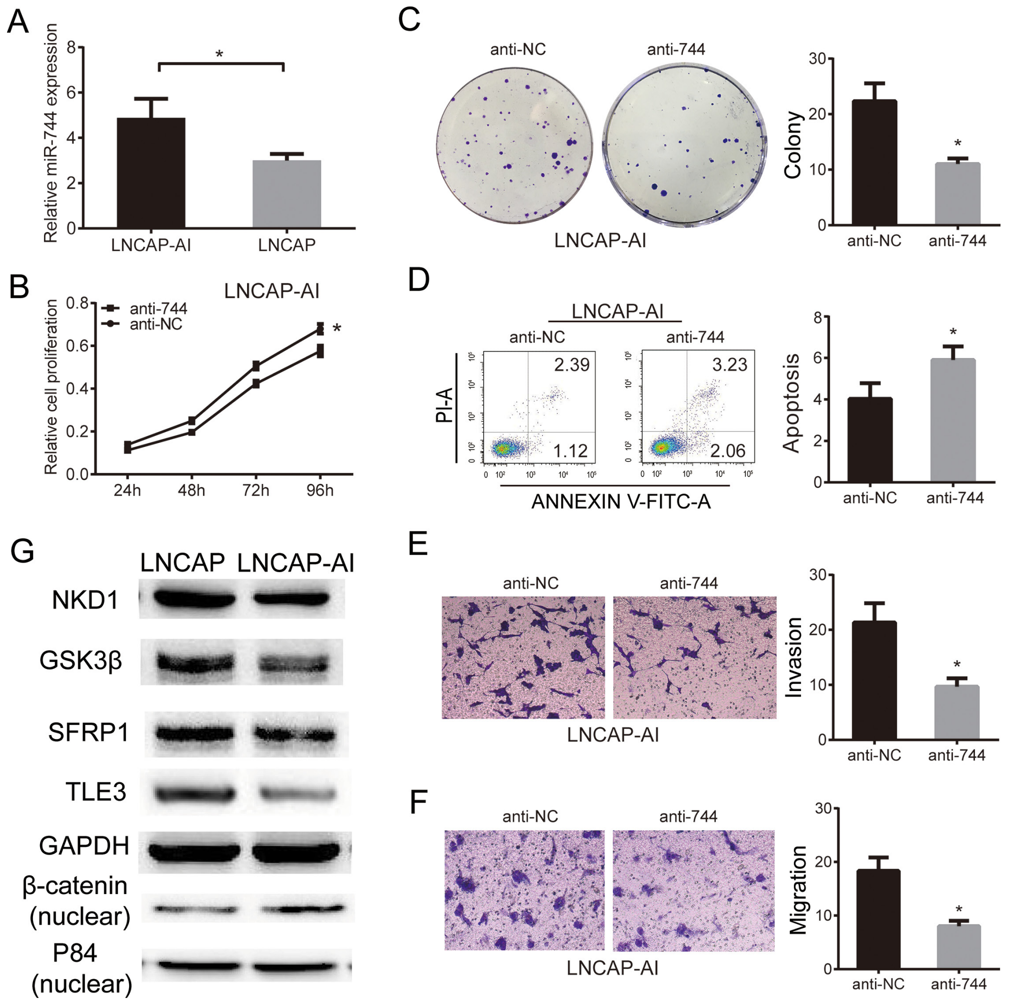

Supplementary Figure 1: Reduction of MiR-744 suppresses cells proliferation, migration, and invasion, but promotes apoptosis in PCa cells. (**A**) The expression of miR-744 was significantly up-regulated in androgen-independent subline form LNCaP cells (LNCaP-AI) than LNCaP cells. (*$P < 0.05$). U6 RNA was measured as an internal control. (**B**) MTT assay showed that anti-miR-744 oligos (groups of anti-miR-744) suppressed growth rate in LNCaP-AI cells. (**C**) Colony formation assay indicated that colony number of LNCaP-AI cells transfected with anti-miR-744 oligos was lower than control. (**D**) Cell apoptosis assay. The consequence showed LNCaP-AI cells with anti-miR-744 oligos demonstrate a higher apoptosis than control. (**E**) and (**F**) The results of Transwell assay showed that migration and invasion ability of anti-miR-744 oligos group was lower than negative control in LNCaP-AI cells. Each bar represents the mean ± SD of three independent experiments. *$P < 0.05$. (**G**) Protein expression of NKD1, GSK3β, SFRP1, TLE3 and nuclear β-catenin in LNCaP-AI cells and LNCaP cells was determined by Western blot assay. GAPDH was used as an internal control as well as P84 was served as an internal control in nucleus.

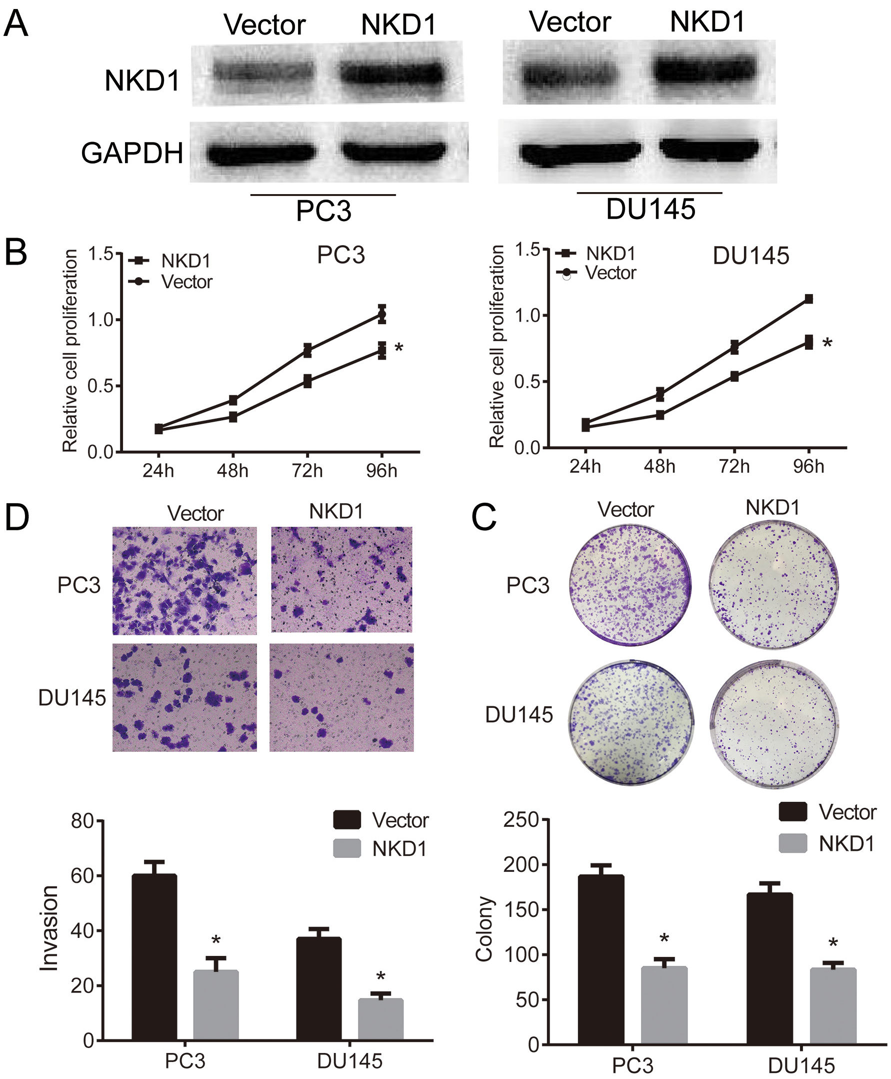

Supplementary Figure 2: Overexpression of NKD1 elicited similar effects on PCa cells with knockdown miR-744. (**A**) Western blot assay showed that overexpressing NKD1 vectors (groups of NKD1) increased the expression of NKD1 protein in PC3 and DU145 cells. (**B**) MTT assay showed that overexpressing NKD1 vectors suppressed growth rate in PC3 and DU145 cells. (**C**) and (**D**) Colony formation assay and Transwell assay showed that overexpression of NKD1 in PC3 and DU145 cells markedly inhibited cell proliferation and colony formation as well as invasive abilities, compared to the control-treated cells. The GFP vector (group of Vector) was used for control. Each bar represents the mean ± SD of three independent experiments. $^*P < 0.05$.

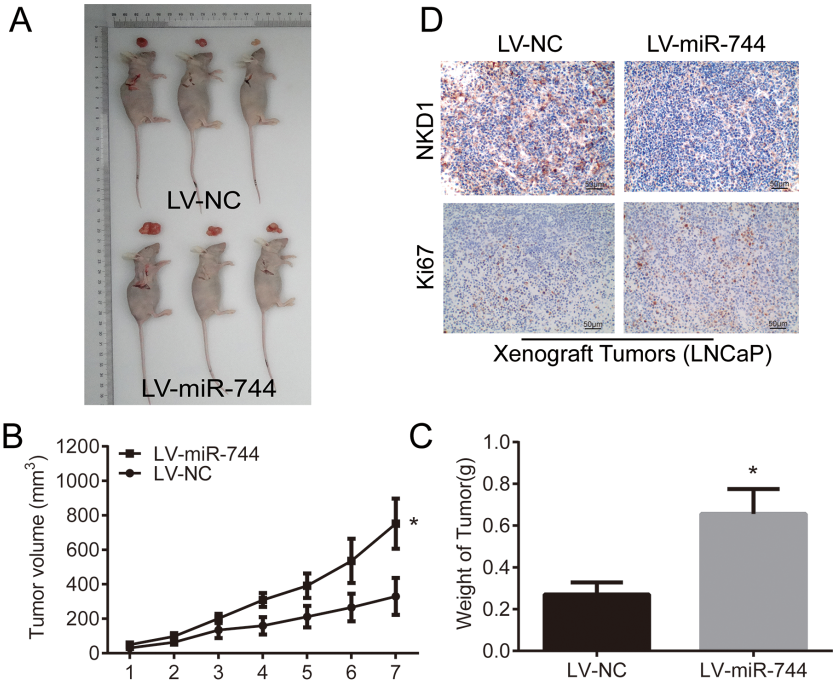

Supplementary Figure 3: Overexpression of MiR-744 promotes the formation of prostate xenograft tumors *in vivo.* (**A**) Subcutaneous tumors formed in nude mice by LNCaP cells stably overexpression of miR-744 or control at 28 days (n = 3/group). (**B**) Tumor formation growth curves after transfection of indicated cells. (**C**) Histograms describing the mean tumor weights of each group. Mean tumor volumes are plotted. (**D**) Immunohistochemical staining of NKD1 and Ki67 in the endpoint tumors revealed that reduced NKD1-positive cells and significantly increased Ki67-positive cells in miR-744 overexpressing LNCaP tumors. Scale bars represent 50 µm. Each bar represents the mean ± SD of three independent experiments. *P < 0.05.

Supplementary Table 1: Microarry analysis in ADPC and CRPC

High expressed miRNA	Normalized Intensity		CV		Log2(Ratio)	*P*-value
	CRPC	ADPC	CRPC	ADPC	ADPC/CRPC	ADPC/CRPC
MiR-30c-1	25610.696	1082.053	0.017	0.029	−4.565	7.17E–8
MiR-3945	10629.050	633.396	0.020	0.018	−4.068	0
MiR-744	16636.523	1127.638	0.066	0.517	−3.883	1.70E–5
MiR-1292	3847.328	281.910	0.250	0.026	−3.781	3.10E–3
MiR-4635	7676.017	575.815	0.046	0.045	−3.737	4.00E–6

Supplementary Table 2: Cox regression analysis of prognostic factors in PCa patients

Name	Hazard Ratio (HR)	95% CI for HR		*P* value
		Lower	Upper	
MiR-744	8.273	1.847	37.060	0.006
GS	4.794	1.817	12.645	0.002
PSA	1.047	1.014	1.081	0.005
LNI	0.033	0.004	0.282	0.002
Age	0.924	0.836	1.022	0.124
SMS	1.623	0.363	7.257	0.526
ECE	1.155	0.211	6.330	0.868
SVI	5.881	0.746	46.361	0.093
pStage	0.914	0.162	5.176	0.919

CI, confidence interval. GS, gleason score. PSA, prostate-specific antigen. LNI, lymph node invasion. SMS, surgical margins. ECE, extracapsular extension. SVI, seminal vesicle invasion. pStage, pathological stage.

Supplementary Table 3: Differential expression genes in Wnt signaling by knockdown miR-744

KEGG Wnt Signaling Pathway	Regulation	Fold change	Log(Fold change)
MAPK9	up	1.61	0.69
NKD1	up	1.54	0.63
CSNK2α1	up	1.51	0.59
MAPK8	down	−2.17	−1.12
CACYBP	down	−1.54	−0.63
FBXW11	down	−1.52	−0.61
